# The lncRNA CRNDE promotes colorectal cancer cell proliferation and chemoresistance via miR-181a-5p-mediated regulation of Wnt/β-catenin signaling

**DOI:** 10.1186/s12943-017-0583-1

**Published:** 2017-01-13

**Authors:** Peng Han, Jing-wen Li, Bo-miao Zhang, Jia-chen Lv, Yong-min Li, Xin-yue Gu, Zhi-wei Yu, Yun-he Jia, Xue-feng Bai, Li Li, Yan-long Liu, Bin-bin Cui

**Affiliations:** Department of Colorectal Surgery, The Affiliated Tumor Hospital of Harbin Medical University, 150 Haping Road, Harbin, 150040 People’s Republic of China

**Keywords:** CRNDE, miR-181a-5p, Wnt/β-catenin signaling, Colorectal cancer, Proliferation, Chemoresistance

## Abstract

**Background:**

With more than 600,000 mortalities each year, colorectal cancer (CRC) is the third most commonly diagnosed type of cancer worldwide. Recently, mechanisms involving noncoding RNAs have been implicated in the development of CRC.

**Methods:**

We examined expression levels of lncRNA CRNDE and miR-181a-5p in 64 cases of CRC tissues and cell lines by qRT-PCR. Gain-of-function and loss-of-function assays were performed to examine the effect of CRNDE and miR-181a-5p on proliferation and chemoresistance of CRC cells. Using fluorescence reporter and western blot assays, we also explored the possible mechanisms of CRNDE in CRC cells.

**Results:**

In this study, we found that the expression levels of the CRNDE were upregulated in CRC clinical tissue samples. We identified microRNA miR-181a-5p as an inhibitory target of CRNDE. Both CRNDE knockdown and miR-181a-5p overexpression in CRC cell lines led to inhibited cell proliferation and reduced chemoresistance. We also determined that β-catenin and TCF4 were inhibitory targets of miR-181a-5p, and that Wnt/β-catenin signaling was inhibited by both CRNDE knockdown and miR-181a-5p overexpression. Significantly, we found that the repression of cell proliferation, the reduction of chemoresistance, and the inhibition of Wnt/β-catenin signaling induced by CRNDE knockdown would require the increased expression of miR-181a-5p.

**Conclusions:**

Our study demonstrated that the lncRNA CRNDE could regulate the progression and chemoresistance of CRC via modulating the expression levels of miR-181a-5p and the activity of Wnt/β-catenin signaling.

**Electronic supplementary material:**

The online version of this article (doi:10.1186/s12943-017-0583-1) contains supplementary material, which is available to authorized users.

## Background

With more than 600,000 mortalities each year, colorectal cancer (CRC) is the third most commonly diagnosed type of cancer worldwide [1, 2]. Like many other cancer types, the development of CRC is a process characterized by multiple stages and involves accumulation of both genetic and epigenetic changes [3]. An extensive body of recent research has revealed that the initiation and progression of CRC are regulated by a number of cellular signaling pathways, including the TGFβ/SMAD pathway, the p53 pathway, the EGFR/MAPK pathway, the PI3K-Akt pathway, and the Wnt/β-catenin pathway [4]. Recently, mechanisms involving noncoding RNAs have also been implicated in the development of CRC [5–8].

Based on size, noncoding RNAs (ncRNAs) can be categorized into small ncRNAs (<200 nt) and long ncRNAs (>200 nt). Small ncRNAs, including microRNAs, have been demonstrated to function primarily via regulating gene expression, including modulating mRNA stability and repressing protein translation [9]. A number of microRNAs were found to act as oncogenes or tumor suppressor genes by previous reports [10–12]. Characterized by lack of cross species conservation [13, 14], the mechanism of action for long ncRNAs (lncRNAs) is poorly understood. However, rapidly accumulating evidence has consistently indicated that lncRNAs play essential roles in a variety of cellular processes, and therefore may contribute to the development of cancer and other human diseases [15, 16].

The lncRNA Colorectal Neoplasia Differentially Expressed (CRNDE) is transcribed from chromosome 16 on the strand opposite to the adjacent IRX5 gene [17]. The expression levels of CRNDE were originally found to be increased in CRC [17], and were also upregulated in a number of solid and hematopoietic cancer types, including liver [18], pancreatic [19], ovarian [20], kidney cancer [21], glioma [22], and leukemia [23]. Besides cancer progression, the CRNDE gene also participated in the regulation of neuronal differentiation, gametogenesis and other developmental processes [24]. In addition, the mouse ortholog of CRDNE was demonstrated to be involved in the maintenance of cellular pluripotency [25]. Although there was some preliminary evidence that CRNDE could interact with chromatin-modifying complexes to affect epigenetic regulation of gene expression [26], the mechanism by which CRNDE plays its role, particularly in the development of CRC, has not yet been elucidated.

In the present study, we found that the expression levels of the lncRNA CRNDE were upregulated in CRC clinical tissue samples. We identified microRNA miR-181a-5p as a target for CRNDE. The expression of miR-181a-5p was inhibited by CRNDE. Knockdown of CRNDE and overexpression of miR-181a-5p in CRC cell lines both led to inhibited cell proliferation and reduced chemoresistance. We also determined that β-catenin and TCF4 were inhibitory targets of miR-181a-5p, and that Wnt/β-catenin signaling was inhibited by both CRNDE knockdown and miR-181a-5p overexpression. Significantly, we found that the repression of cell proliferation, the reduction of chemoresistance, and the inhibition of Wnt/β-catenin signaling in CRC cells induced by CRNDE knockdown all required the activity of miR-181a-5p. Taken together, our study demonstrated that the lncRNA CRNDE could regulate the progression and chemoresistance of CRC via modulating the expression levels of miR-181a-5p and the activity of the Wnt/β-catenin signaling pathway.

## Methods

### Clinical samples

The procedures of this study were approved by the Institutional Review Board of Harbin Medical University. Written informed consent was obtained from 64 participants. Fresh colorectal cancer tissues and paired adjacent normal specimens were collected by surgical resection. The patients with colorectal cancer had received neither chemotherapy nor radiotherapy prior to section. Pathological diagnostics for colorectal cancer were determined by three pathologists. The clinical characteristics in 64 CRC patients is presented in the Table 1.

**Table 1:** Correlation between CRNDE expression and clinical characteristics of CRC

Factor	Number	CRNDE expression		*P*
		Low	High	
Age (y)				0.117
<60	23	15	8	
≥60	41	17	24	
Gender				0.123
Male	39	16	23	
Female	25	16	9	
Tumor location				0.617
Colon	31	14	17	
Rectum	33	18	15	
Tumor size (cm)				0.002**
< 5	29	21	8	
≥ 5	35	11	24	
Histologic grade				0.001**
Grade 1 + 2	51	31	20	
Grade 3 + 4	13	1	12	
Lymph node metastasis				0.011*
Yes	37	13	24	
No	27	19	8	
Distant metastasis				0.750
M0	52	27	25	
M1	12	5	7	

*P < 0.05, **P < 0.01

### TCGA analysis

The RNA-seq data of 157 tumor and 30 matched normal samples were downloaded from The Cancer Genome Atlas (TCGA) Data Portal (https://tcga-data.nci.nih.gov/tcga/). The expression of lncRNAs was quantified by the customized data analysis pipeline that including the steps of quality control, alignment, and expression quantification. We utilized UCSC hg19 as the Homo sapiens reference genome and gene model for read mapping and quantification. The correlation analysis was performed based on Pearson product-moment correlation coefficient.

### Cell culture and treatment

Human CRC cell lines HCT116 and SW480 were cultured in Dulbecco’s Modified Eagle Medium with 4.5 g/L glucose (DMEM; Gibco BRL, Grand Island, NY, USA) containing 10% fetal bovine serum (FBS; Gibco BRL) and 1% antibiotic/antimycotic solution. Cells were maintained at 37 °C in an atmosphere of 5% CO_2_ and 95% room air.

LncRNA CRNDE siRNA, control siRNA, miR-181a-5p and miR-181a-5p inhibitor were all obtained from GenePharma (Shanghai, China). 5-fluorouracil (5-Fu) and oxaliplatin (Oxa) were all purchased from Sigma Aldrich (St Louis, MO, USA). Lipofectamine™ 3000 (Life Technologies, San Diego, CA, USA) was used for cell transfection according to manufacturer’s instructions. After 48 h of transfection, cells were treated different concentration of 5-Fu (0, 3, 6, 12, 24 and 48 μg/ml) or Oxa (0, 4, 8, 16, 32 and 64 μg/ml).

### qRT-PCR

Total RNA was extracted from colorectal cancer cell lines and patient specimens using TRIzol reagent (Life Technologies) according to the manufacturer’s manual. One microgram of total RNA was used as template for cDNA synthesis using a PrimeScript RT Reagent Kit with cDNA Eraser (Takara Biotech, Dalian, China) and qRT-PCR was performed using SYBR Premix Ex Taq (Takara Biotech). MiRNA expression were performed in triplicate using SYBR PrimeScriptTM miRNA RT-PCR Kit (Takara Biotech). All qRT-PCR assays was performed on an ABI 7900 system (Applied Biosystems, Foster City, CA, USA). Expression levels of genes or miRNA were normalized to that of the housekeeping gene glyceraldehyde 3-phosphate dehydrogenase (GAPDH) or SNORD6 (U6 snRNA). 2^−ΔΔCt^ method was applied for calculation of relative levels of genes and miRNA expression.

### RNA fluorescence *in-situ* hybridization (FISH)

RNA FISH assays were performed to observe CRNDE location. CRC cells were fixed by 4% formaldehyde for 10 min at room temperature and then permeablized using 0.5% Triton X-100 for 30 min. Afterwards, the cells were washed 3 × for 5 min in PBS and then Hybridized with cDNA probe labeled fluorochromes Cy3 (green).

### 3-(4,5-dimethylthiazol-2-yl)-2,5-diphenyltetrazolium bromide (MTT) assay

MTT assay was used for cell proliferation and cell inhibition rate analysis. Colorectal cancer cells were seeded in 96-well plate at a density of 1 × 10^3^ cells per well. After treatment, the cells were washed twice with phosphate buffer saline (PBS). Then, 10 μL of MTT dye (5 mg/mL) was added to the wells at different time points. After 4 h incubation, 100 μL of dimethyl sulfoxide (DMSO) was added to each well to dissolve the formazan crystals and the absorbance was measured at 590 nm.

### Colony formation assay

HCT116 and SW480 cells (0.5 × 10^3^ cells per well) were seeded in a six-well plate and cultured for 10 days after treatment. Colonies were then fixed with 10% formaldehyde for 10 min and stained for 5 min with 0.5% crystal violet. Then the number of colonies was counted using ImageJ and images were taken under Olympus microscope (Tokyo, Japan).

### Bromodeoxyuridine (BrdU) assay

Colorectal cancer cell proliferation was determined by BrdU assay using a BrdU kit (Abcam, Cambridge, MA, USA) according to the manufacturer’s instructions. Cells were growing on cover slips and incubated with BrdU during DNA synthesis for 1 h followed by staining with an anti-BrdU antibody after treatment. Images were acquired using an Olympus camera under a microscope.

### Establishment of 5-Fu resistant cells

5-Fu resistant colorectal cells were generated by continuous exposure to increasing concentrations of 5-Fu (from 5 to 30 μg/ml) with repeated subculture until fully resistant to 5-Fu. Cells were first cultured in growing medium with 5 μg/ml 5-Fu for two months and the concentration of 5-Fu increased 5 μg/ml every two months.

### Luciferase assay

CRNDE wild type with potential miR-181a-5p binding sites or mutant of each sites were generated and fused to the luciferase reporter vector psi-CHECK-2 (Promega, Madison, WI, USA). The full-length wild-type (WT) 3′ untranslated region (UTR) containing the predicted miR-181a-5p targeting site, and mutant (MUT) 3′-UTR of β-catenin and TCF4 were amplified and cloned into the psi-CHECK-2 vector. HEK293T cells were placed on a 24-well plate and grew till 80% confluence. Cells were then co-transfected with luciferase plasmids and miR-181a-5p or control miRNA. After 48 h transfection, firefly and renilla luciferase activities were measured with a Dual-Luciferase Reporter Assay System (Promega).

### Pull-down assays

Pull-down assays were performed as described previously [27]. Briefly, S1-CRNDE and S1-CRNDE mutant were generated, and cotransfected with or without miR-181a-5p inhibitor. After 48 h of transfection, cells were harvested and washed by PBS for two times, then crosslinked in 0.37% formaldehyde, incubated in ice-cold lysis buffer (150 mM NaCl, 10 mM Hepes, 3 mM MgCl_2_, 10% glyceral, 1% NP-40, 2 mM DTT, 1 mM PMSF, 1 × protenase inhibitor (Sigma), 10 ul RNase inhibitor (promega)). The cell lysate was precleaned in agarose beads (Santa Cruz Biotechnology) at 4 °C for 1 h, then incubated and rotated in streptavidin beads (Thermo Fisher Scientific, San Jose, CA, USA) at 4 °C for 3 h. The streptavidin beads were collected, washed in elution buffer (50 mM Hepes, 5 mM EDTA, 100 mM NaCl, 1% SDS, 10 mM DTT). The beads were heated at 70 °C for 45 min. miR-181a-5p expression was examined by qRT-PCR.

### Western blot analysis

Total proteins were prepared from colorectal cells using RIPA buffer (50 mm Tris-HCl, 150 mm NaCl, 1 mm EDTA, 0.1% SDS, 1% Triton X-100, 0.1% sodium deoxycholate) with proteinase inhibitor cocktail (Boster, Wuhan, China). The lysates were centrifuged at 12,000 rpm for 15 min at 4 °C and protein concentration was measured by BCA kit (Beyotime Biotechnology, Beijing, China). Equal quantities of protein were electrophoresed through a 10% sodium dodecyl sulfate/polyacrylamide gel and transferred to a nitrocellulose membranes (Millipore, Billerica, MA). The membranes were blocked and then incubated with β-catenin, TCF4, Cyclin D1, Axin2 and GAPDH (Santa Cruz Biotechnology) overnight at 4 °C. Subsequently, the membranes were incubated with a HRP-conjugated anti-mouse or -rabbit secondary antibody (Santa Cruz Biotechnology) at room temperature for 1 h. The protein bands were visualized using a chemiluminescence reagent (ECL) kit (Beyotime Biotechnology).

### Statistics

The data are expressed as the mean ± standard error (SD) from at least three independent experiments. The differences between two groups were analyzed using Student’s t test or a one-way ANOVA when more than two groups were compared. Correlations between miRNA expression and its targets were analyzed by the Spearman’s test. A value of *P* < 0.05 was considered statistically significant. All statistial analyses were performed using GraphPad software version 5.0 (GraphPad Software, CA, USA).

## Results

### CRNDE expression is elevated in CRC tissue

**Fig. 1:**
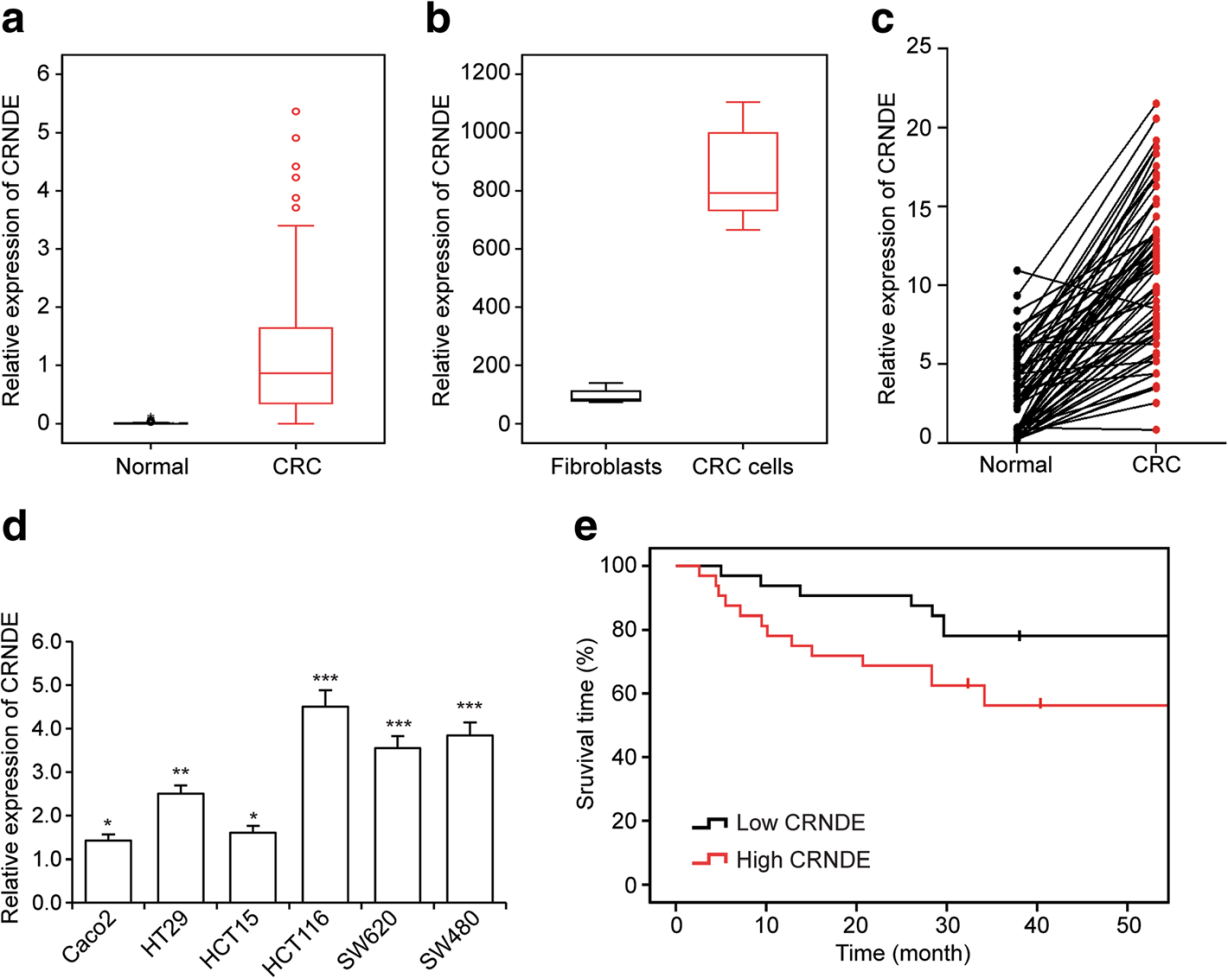
CRNDE expression is elevated in CRC tissue. **a** Expression levels of CRNDE analyzed from data retrieved from the TCGA database in CRC patients (*P* <0.001). **b** Expression levels of CRNDE analyzed from GSO/GDS4385 (*P* < 0.01). **c** Expression levels of CRNDE as determined by qRT-PCR in 64 clinical CRC samples compared to those in adjacent normal tissue samples (*P* < 0.001). **d** CRNDE expression in six CRC cell lines compared with normal colorectal tissue pool. *P < 0.05, ***P < 0.01 and ***P < 0.001. **e** Kaplan-Meier analysis of the association between GRIM-19 expression and the survival in 64 clinical CRC patients. *P* < 0.001

In order to investigate the relevance of lncRNA CRNDE in CRC development, we first sought to determine the levels of CRNDE expression form TCGA database. As shown in Fig. 1a, expression of CRNDE was significantly upregulated in CRC tissues (*P* < 0.001). We also complied gene expression data from the GSO/GDS4385 database, and confirmed that the expression levels of CRNDE were increased in CRC tissue (Fig. 1b, *P* < 0.001). Next, CRNDE expression was examined by qRT-PCR on samples from 64 clinical CRC patients compared to adjacent normal tissue and elevated CRNDE expression was observed (Fig. 1c, *P* < 0.001). We then performed qRT-PCR to analyze CRNDE expression in six CRC cell lines (Caco2, HT29, HCT15, HCT116, SW620 and SW480). As compared to 3 normal CRC sample, overexpressed levels of CRNDE expression was seen in all CRC cell line (Fig. 1d). Furthermore, we performed Kaplan-Meier survival analysis of the association between CRNDE expression and the survival in 64 clinical CRC patients. As shown in Fig. 1e, high expression of CRNDE significantly shorterned CRC patient survival time. We next detected CRNDE location by RNA FISH assays and found nuclear location of CRNDE in CRC cells (Additional file 1). These results were consistent with previous findings in another cohort of patients [17], and suggested that CRNDE might be involved in the regulation of CRC development.

### CRNDE binds to miR-181a-5p and represses its expression

**Fig. 2:**
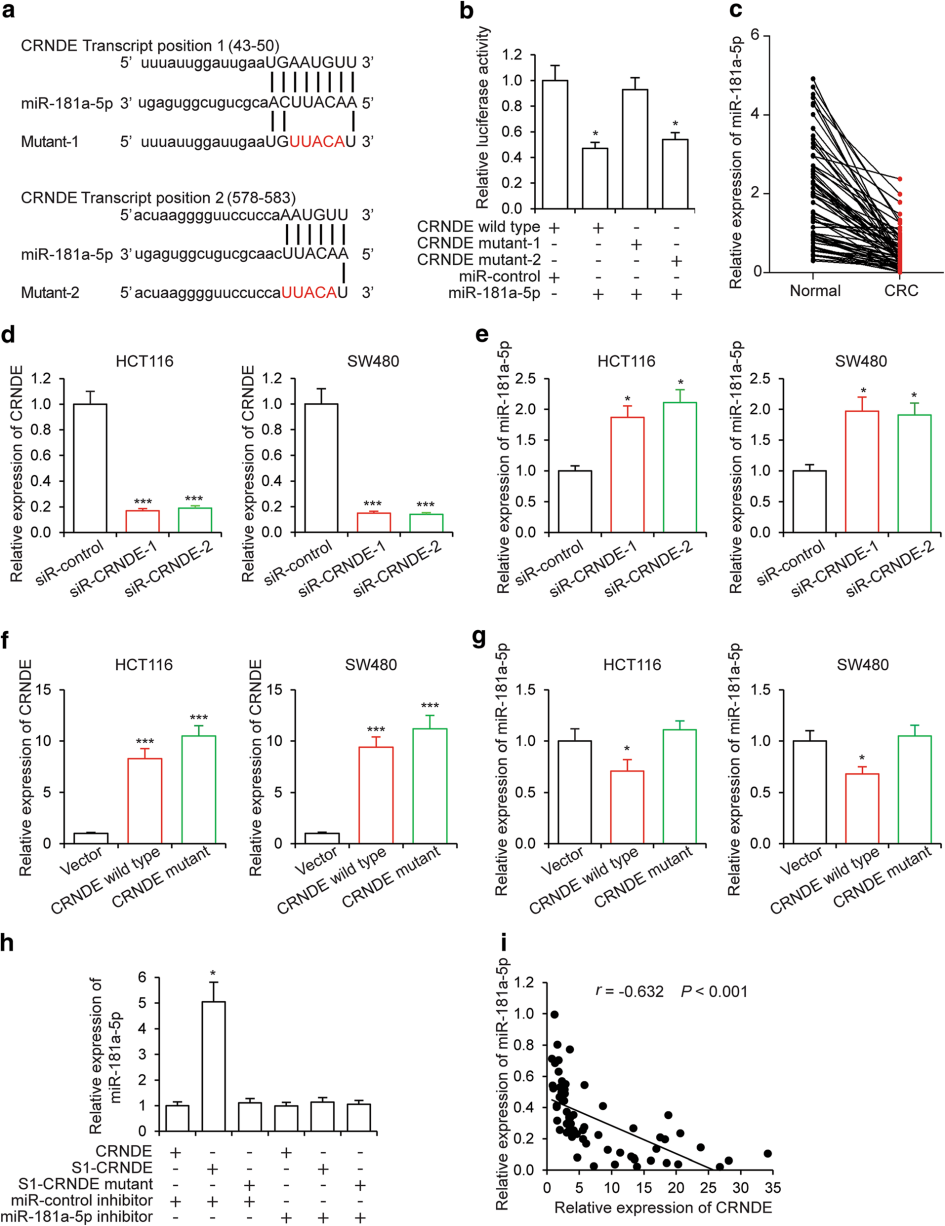
CRNDE binds to miR-181a-5p and represses its expression. **a** Schematic illustration of the predicted binding sites between CRNDE and miR-181a-5p, and mutation of potential miR-181a-5p binding sequence in CRNDE. **b** Luciferase assays in 293 T cells transfected CRNDE wild type or mutants with miR-181a-5p (*P < 0.05). **c** Expression levels of miR-181a-5p as determined by qRT-PCR in 64 CRC samples compared to those in adjacent normal tissue samples (*P* < 0.001). **d** Expression levels of CRNDE and **e** miR-181a-5p as determined by qRT-PCR in HCT116 and SW480 cells transfected with siRNA targeting CRNDE (siR-CRNDE) or a control siRNA (siR-control) (*P < 0.05, ***P < 0.001). **f** Expression levels of CRNDE and **g** miR-181a-5p as determined by qRT-PCR in HCT116 and SW480 cells transfected with plasmids overexpressing wild type, mutant CRNDE or an empty vector (*P < 0.05, ***P < 0.001). **h** HEK293T were transfected with CRNDE, S1-CRNDE or S1-CRNDE mutant with or without miR-181a-5p inhibitor, then pull-down assays were performed (*P < 0.05). **i** Inverse correlation between the expression levels of CRNDE and those of miR-181a-5p in 64 CRC samples (*r* = −0.632, *P* < 0.001)

To determine the mechanism of action for CRNDE in CRC development, we identified miR-181a-5p as a potential target of CRNDE with LncBase Predicted v.2 of DIANA tools. There are two putative binding sites of CRNDE at the regions of 43–35 and 578–583 on miR-181a-5p (Fig. 2a). We generated wild type CRNDE luciferase plasmids containing potential miR-181a-5p binding sites and their mutant of each site. These plasmids was co-transfected with miR-181a-5p into HEK293T cells, respectively, and then luciferase assays were performed. As shown in Fig. 2b, miR-181a-5p could reduce wild type and mutant-2 CRNDE luciferase activity. But it can’t affect mutant-1 activity. The results indicated that miR-181a-5p binds transcript position (43–50) of CRNDE. In tissue samples from 64 clinical CRC patients, we determined that the expression levels of miR-181a-5p were significantly downregulated (Fig. 2c), a trend opposite to that of CRNDE. To examine whether miR-181a-5p was indeed a target of CRNDE, we first knocked down CRNDE expression in two independent CRC cell lines, HCT116 and SW480 cells, by siRNA transfection. The efficacy of knockdown was confirmed by qRT-PCR analysis (Fig. 2d). In both CRNDE knockdown cell lines, we observed elevated expression levels of miR-181a-5p (Fig. 2e). We next increased wild type and mutant CRNDE expression in CRC cell lines (Fig. 2f), we found that the expression levels of miR-181a-5p were significantly inhibited by wild type CRNDE but not by mutant (Fig. 2g). Importantly, overexpression (Additional file 2a) or knockdown (Additional file 2b) of miR-181a-5p did not cause any change in the expression levels of CRNDE (Additional file 2c and d), indicating that miR-181a-5p was downstream of CRNDE. To further investigate whether CRNDE and miR-181a-5p binding together, we performed pull-down assays. As shown in Fig. 2h, CRNDE was confirmed binding with miR-181a-5p and this binding was inhibited by miR-181a-5p inhibitor. Mutation of CRNDE binding sequence with miR-181a-5p inhibited miR-181a-5p precipitation. The inhibitory regulation of miR-181a-5p by CRNDE was further corroborated by the analysis of clinical samples. We also observed inverse correlation between the expression levels of CRNDE and miR-181a-5p in CRC tissue samples (Fig. 2i, *r* = −0.632, *P* < 0.001). Taken together, our data supported that miR-181a-5p was an inhibitory target of CRNDE in both CRC cells and tissue samples.

### CRNDE promotes CRC cell proliferation and chemoresistance

**Fig. 3:**
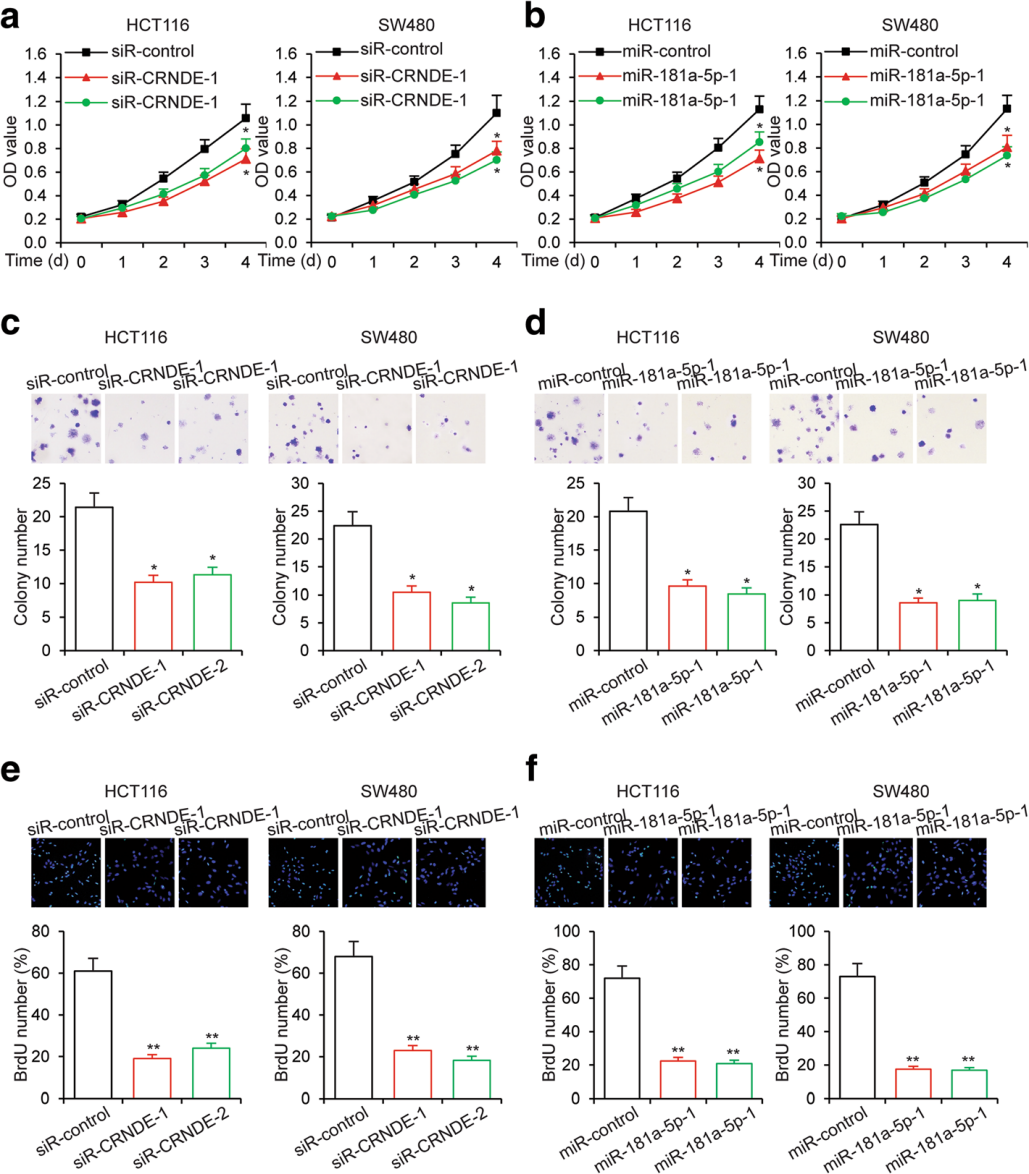
CRNDE knockdown and miR-181a-5p overexpression inhibit CRC cell proliferation. **a** MTT cell proliferation assays performed in HCT116 and SW480 cells transfected with siR-CRNDE or siR-control, or in HCT116 and SW480 cells transfected with miR-181a-5p or a control microRNA (miR-control). **b** colony formation assays performed in HCT116 and SW480 cells transfected with siR-CRNDE or siR-control, or in HCT116 and SW480 cells transfected with miR-181a-5p or miR-control. **c** BrdU assay performed in HCT116 and SW480 cells transfected with siR-CRNDE or siR-control, or in HCT116 and SW480 cells transfected with miR-181a-5p or miR-control. *P < 0.05, **P < 0.01

To investigate the function of CRNDE on CRC cell proliferation, we first knocked down the expression of CRNDE in two CRC cell lines, HCT116 and SW480 cells. We then performed MTT cell proliferation assay on these cells. We found that knockdown of CRNDE in both cell lines significantly inhibited cell proliferation (Fig. 3a). Significantly, overexpression of miR-181a-5p in both HCT116 and SW480 cells was able to recapitulate the inhibitory effects on cell proliferation conferred by CRNDE knockdown (Fig. 3b). In contrast, CRNDE overexpression and miR-181a-5p knockdown both led to increased cell proliferation in CRC cells (Additional file 3a and b). We also performed additional assays to confirm the regulation of cell proliferation by CRNDE and miR-181a-5p. In crystal violet staining assay, colony formation of CRC cells was inhibited by knockdown of CRNDE (Fig. 3c) and by overexpression of miR-181a-5p (Fig. 3d). Consistently, we observed significantly less actively divided cells in BrdU assay with CRNDE knockdown (Fig. 3e) and miR-181a-5p overexpression (Fig. 3f). These data collectively indicated that CRNDE promoted CRC cell proliferation.

**Fig. 4:**
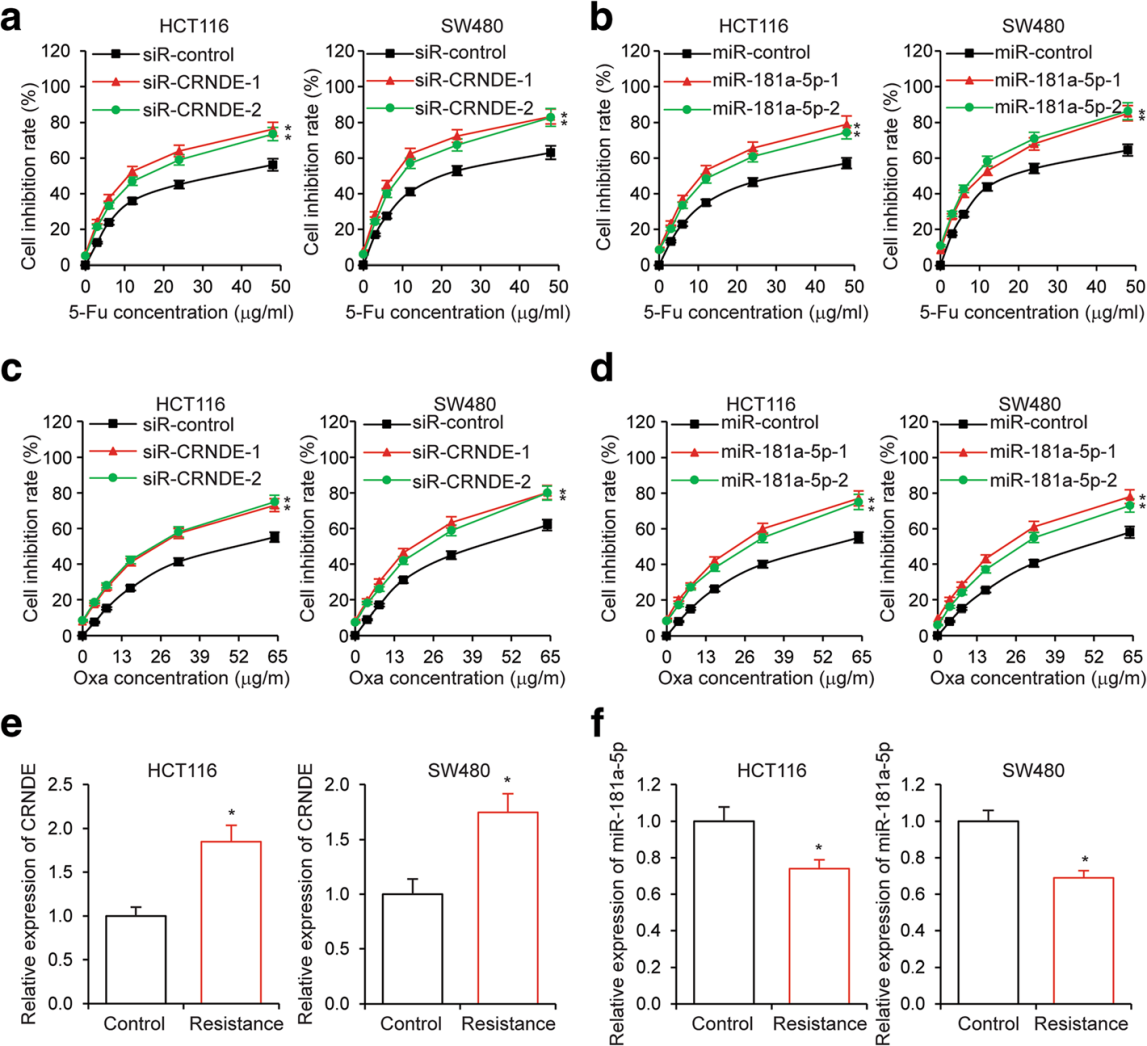
CRNDE knockdown and miR-181a-5p overexpression repress CRC cell chemoresistance. **a** MTT cell proliferation assay performed in HCT116 and SW480 cells transfected with siRNA targeting CRNDE or a control siRNA and treated with the indicated concentrations of 5-Fu. **b** MTT cell proliferation assay performed in HCT116 and SW480 cells transfected with miR-181a-5p or a control microRNA and treated with the indicated concentrations of 5-Fu. **c** MTT cell proliferation assay performed in HCT116 and SW480 cells transfected with siR-CRNDE or a control siRNA and treated with the indicated concentrations of Oxa. **d** MTT cell proliferation assay performed in HCT116 and SW480 cells transfected with miR-181a-5p or miR-control and treated with the indicated concentrations of Oxa. **e** Expression levels of CRNDE and **f** miR-181a-5p as determined by qRT-PCR in 5-Fu resistant HCT116 and SW480 cells. *P < 0.05

As previous research demonstrated that noncoding RNAs are involved in the chemoresistance of cancer cells [28], we hypothesized that CRNDE and miR-181a-5p could also regulate the chemoresistance of CRC cells. To test this hypothesis, we treated CRNDE knockdown or miR-181a-5p overexpressing CRC cells with an increasing serial of concentrations of 5-Fu. As expected, 5-Fu led to cell growth inhibition in a dose-dependent manner. We found that CRNDE knockdown (Fig. 4a) and miR-181a-5p overexpression (Fig. 4b) appeared to increase the sensitivity of CRC cells to 5-Fu treatment and therefore further inhibited cell growth. Conversely, CRNDE overexpression (Additional file 4a) and miR-181a-5p knockdown (Additional file 4b) resulted in decreased 5-Fu sensitivity, and partially alleviate the growth inhibition of CRC cells induced by 5-Fu treatment. These results were further confirmed by the treatment of another chemotherapy drug Oxa. Consistently, CRNDE knockdown (Fig. 4c) and miR-181a-5p overexpression (Fig. 4d) led to increased sensitivity of CRC cells to Oxa treatment, and CRNDE overexpression (Additional file 4c) and miR-181a-5p knockdown (Additional file 4d) led to decreased Oxa sensitivity of CRC cells. Importantly, we also found that in chemoresistant CRC cells, the expression levels of CRNDE were significantly increased (Fig. 4e), while the expression levels of miR-181a-5p were decreased (Fig. 4f). Taken together, these data indicated that CRNDE increased the resistance of CRC cells against chemotherapy drugs.

### MiR-181a-5p targets β-catenin/TCF4 and inhibits Wnt/β-catenin signaling

**Fig. 5:**
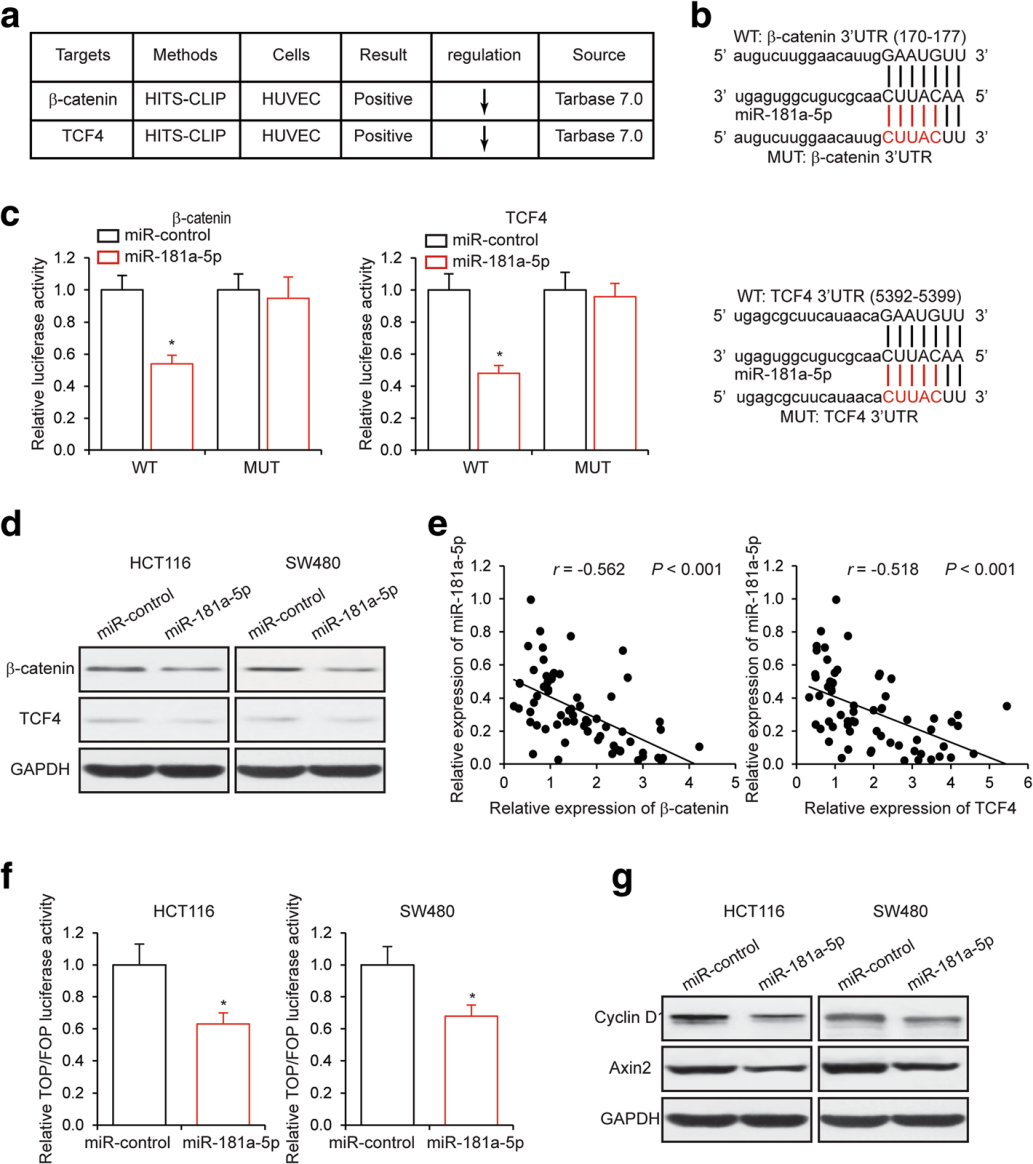
MiR-181a-5p targets β-catenin/TCF4 and inhibits Wnt/β-catenin signaling. **a** Expression levels of β-catenin and TCF4 shown as downregulated in HUVEC overexpressing miR-181a-5p from TarBase v7.0 database of DIANA TOOLS. **b** Schematic illustration of the predicted miR-181a-5p binding sites with the 3′-UTR of β-catenin and TCF4. **c** Luciferase activity assay performed in HEK293 cells co-transfected with miR-181a-5p and luciferase reporter plasmids driven by either wild type (WT) or mutant (MUT) 3′-UTR of β-catenin and TCF4 that was devoid of miR-181a-5p binding activity. **d** The protein levels of β-catenin and TCF4 as determined by Western blot analysis in HCT116 and SW480 cells transfected with miR-181a-5p or miR-control. **e** Inverse correlation between the expression levels of miR-181a-5p and those of β-catenin (*r* = −0.562, *P* < 0.001), and between the expression levels of miR-181a-5p and those of TCF4 (*r* = −0.518, *P* < 0.001) in 64 CRC samples. **f** TOP/FOP luciferase activity in HCT116 and SW480 cells transfected with miR-181a-5p or a miR-control. **g** The protein levels of Cyclin D1 and Axin2 as determined by Western blot analysis in HCT116 and SW480 cells transfected with miR-181a-5p or miR-control. *P < 0.05

We next used publicly available algorithms (DIANA TOOLS and microRNA.org) to identify potential targets of miR-181a-5p. From TarBase v7.0 of DIANA TOOLS [29], we noticed that intracellular signal transducer β-catenin and transcriptional factor TCF4 in the Wnt/β-catenin signaling pathway were downregulated in human umbilical vein endothelial cells (HUVEC) after miR-181a-5p transfection (Fig. 5a). We then sought to confirm this prediction in the context of CRC cells. Based on the predicted binding sites of miR-181a-5p, we generated β-catenin and TCF4 3′-UTR wild type (WT) and mutant (MUT) luciferase reporter plasmids (Fig. 5b). We then performed luciferase reporter assays by co-transfecting luciferase reporter plasmids with miR-181a-5p. We found that the overexpression of miR-181a-5p decreased the luciferase activity driven by the wild type 3′-UTRs of both β-catenin and TCF4, but caused no change in the luciferase activity driven by the mutant 3′-UTRs of both β-catenin and TCF4 (Fig. 5c). In contrast, knockdown of miR-181a-5p increased the luciferase activity driven by the wild type 3′-UTRs of both β-catenin and TCF4, but not by the mutant 3′-UTRs of both β-catenin and TCF4 (Additional file 5a). Furthermore, the protein levels of both β-catenin and TCF4 in CRC cells were reduced by miR-181a-5p overexpression (Fig. 5d), and were increased by miR-181a-5p knockdown (Additional file 5b). The inhibition of β-catenin and TCF4 by miR-181a-5p was also confirmed in clinical samples. In 64 CRC tissue samples, we were able to establish inverse correlation between the expression levels of miR-181a-5p and β-catenin (*r* = −0.562, *P* < 0.001), and between those of miR-181a-5p and TCF4 (*r* = −0.518, *P* < 0.001) (Fig. 5e). Since β-catenin and TCF4 are important components in the Wnt/β-catenin signaling pathway, we next sought to determine whether miR-181a-5p could affect the activity of Wnt/β-catenin signaling. With TOP/FOP luciferase activity assay, we found that overexpression of miR-181a-5p significantly inhibited the activity of Wnt/β-catenin signaling (Fig. 5f), and that knockdown of miR-181a-5p stimulated the activity of Wnt/β-catenin signaling (Additional file 5c). Finally, we found that the levels of downstream target genes of the Wnt/β-catenin signaling pathway, including Cyclin D1 and Axin2, were substantially decreased by miR-181a-5p overexpression in CRC cells (Fig. 5g).

### CRNDE promotes CRC cell proliferation and chemoresistance via miR-181a-5p mediated regulation of Wnt/β-catenin signaling

**Fig. 6:**
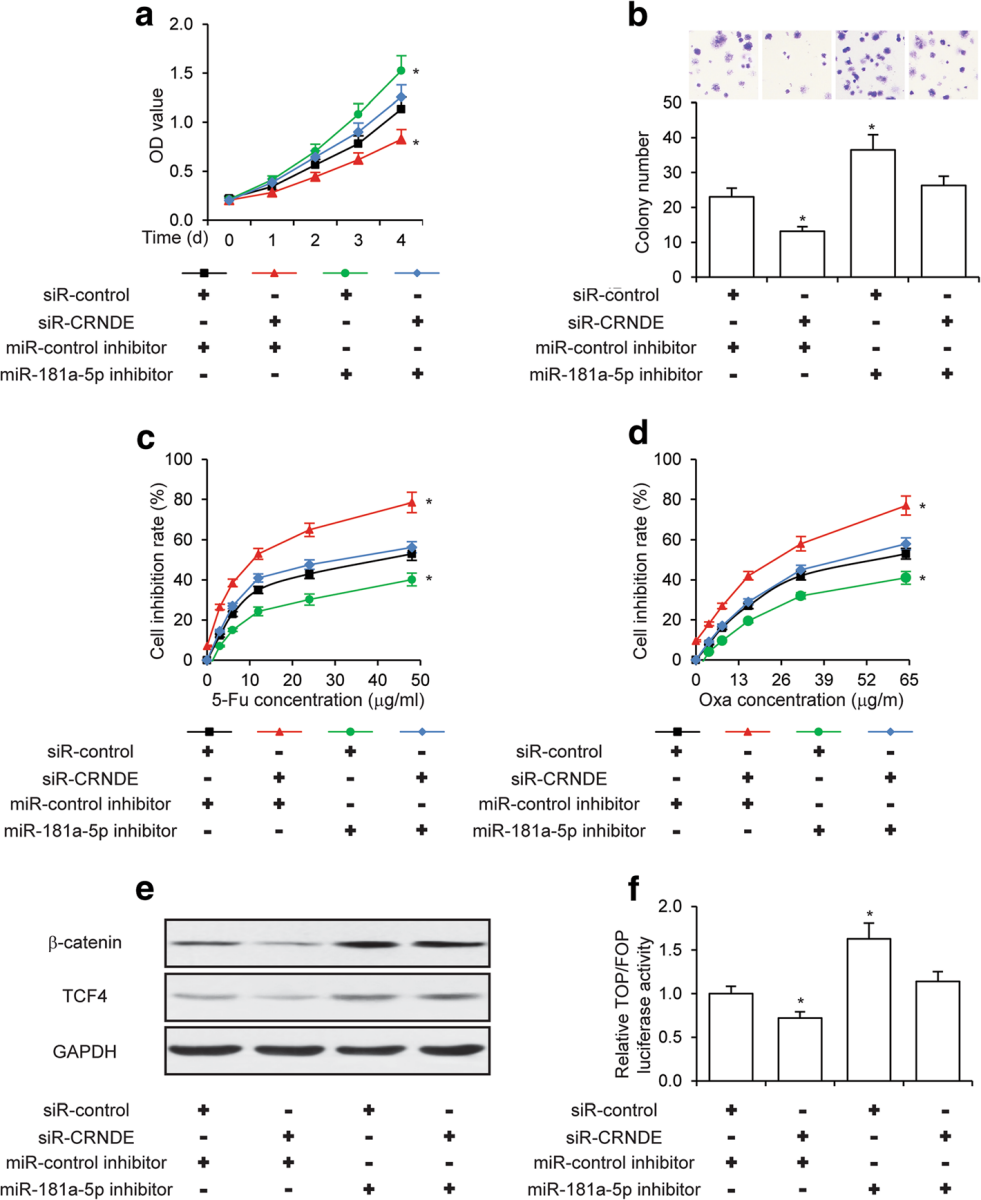
Regulation of CRC cell proliferation, chemoresistance and Wnt/β-catenin signaling by CRNDE requires miR-181a-5p. **a** MTT cell proliferation assay, **b** colony formation assay, **c** MTT cell proliferation assay under the treatment of the indicated concentrations of 5-Fu or **d** Oxa, **e** The protein levels of β-catenin and TCF4 as determined by Western blot analysis, and **f** TOP/FOP luciferase activity assay performed in HCT116 cells transfected with siR-CRNDE or siR-control simultaneously with inhibitors of miR-181a-5p or miR-control. *P < 0.05

**Fig. 7:**
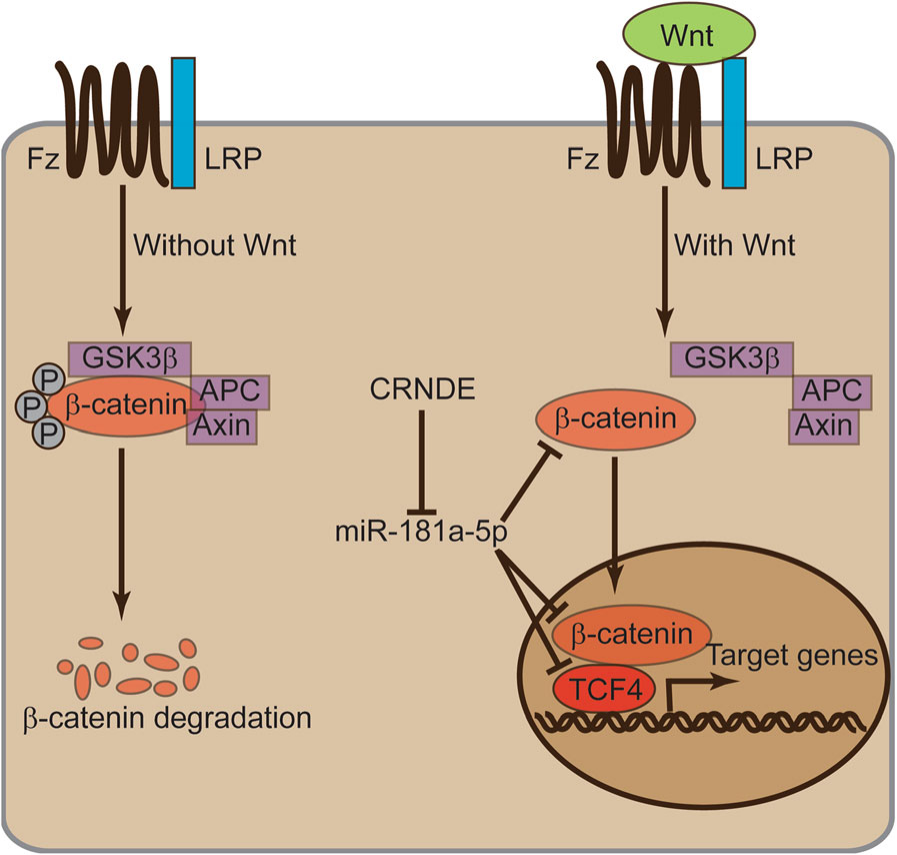
Schematic representation of Wnt/β-catenin signaling involved in regulation of CRNDE and miR-181a-5p

Our previous results demonstrated that CRNDE knockdown and miR-181a-5p overexpression led to inhibition of CRC cell proliferation and chemoresistance. Since CRNDE inhibited miR-181a-5p expression, and miR-181a-5p inhibited Wnt/β-catenin signaling, it is possible that the inhibition of CRC cell proliferation and chemoresistance induced by CRNDE knockdown was due to increased miR-181a-5p expression and consequent inhibition of Wnt/β-catenin signaling. To test this hypothesis, we knocked down β-catenin or TCF4, respectively, and performed MTT cell proliferation assays in the presence of 5-Fu or Oxa. As showed in Additional file 6, inhibition of Wnt/β-catenin signaling resulted in CRC cell chemoresistance. We next concomitantly knocked down the expression of miR-181a-5p in CRNDE knockdown CRC cells. Significantly, while CRNDE knockdown led to significantly inhibited CRC cell proliferation, simultaneous miR-181a-5p knockdown completely reversed the inhibition of cell proliferation (Fig. 6a), indicating that the increased levels of miR-181a-5p expression were essential for the cell proliferation inhibition induced by CRNDE knockdown. Consistently, miR-181a-5p knockdown also completely reversed the inhibition of colony formation of CRC cells caused by CRNDE knockdown (Fig. 6b). These phenotypes were not only observed in HCT116 cells, but also in SW480 cells (Additional file 7a and b). With regard to the regulation of CRC cell chemoresistance by CRNDE, we found that the increased sensitivity of CRC cells to both 5-Fu (Fig. 6c and Additional file 7c) and Oxa (Fig. 6d and Additional file 7d) treatment induced by CRNDE knockdown was completely abolished by simultaneous miR-181a-5p knockdown, indicating that the increased levels of miR-181a-5p expression were also essential for the increased sensitivity of CRC cells to chemotherapy drugs induced by CRNDE knockdown. Importantly, we also sought to determine whether CRNDE regulated Wnt/β-catenin signaling in CRC cells, and whether this regulation was dependent on miR-181a-5p. As expected, knockdown of CRNDE led to decreased protein levels of both for β-catenin and TCF4, presumably via upregulation of miR-181a-5p. However, simultaneous knockdown of miR-181a-5p was able to reverse the repression of β-catenin and TCF4 expression (Fig. 6e and Additional file 7e). In addition, CRNDE knockdown inhibited the activity of Wnt/β-catenin signaling, as evidenced by TOP/FOP luciferase activity, but this inhibition was abolished by simultaneous miR-181a-5p knockdown (Fig. 6f and Additional file 7f), suggesting that CRNDE regulated Wnt/β-catenin signaling in CRC cells via modulating the expression of miR-181a-5p. Collectively, these data strongly support the hypothesis that CRNDE promotes CRC cell proliferation and chemoresistance via miR-181a-5p mediated regulation of Wnt/β-catenin signaling (Fig. 7).

## Discussion

In this study, we focused our research on the regulation of CRC progression and chemoresistance by the lncRNA CRNDE. We started our investigation by examining the expression levels of CRNDE in CRC clinical samples. We found significant increase in CRNDE expression in CRC samples, and this result was also corroborated by the analysis of available data in the TCGA database. In fact, CRNDE has been shown to be upregulated in numerous types of cancers [18–23], but the molecular mechanism of CRNDE in the regulation of cancer progression was not clear.

We identified miR-181a-5p as a target of CRNDE by bioinformatics prediction based on sequence complementarity. Changes in CRNDE expression led to corresponding changes in the expression levels of miR-181a-5p, but changes in miR-181a-5p expression did not affect the expression levels of CRNDE, suggesting that miR-181a-5p was downstream of CRNDE. The inverse correlation between the expression levels of CRNDE and miR-181a-5p in both clinical samples and the TCGA database further validated the target relation between the two non-coding RNAs. To study the functional roles of CRNDE and miR-181a-5p in regulating CRC progression and chemoresistance, we performed experiments in two independent CRC cell lines. This design would ensure that any result in our study would not be restricted to a single line of cells. We found that knockdown of CRNDE led to repressed cell proliferation and reduced chemoresistance, which was consistent with the oncogenic roles of CRNDE revealed by previous studies. Also, the fact that loss of endogenous CRNDE had inhibitory effects on CRC cell proliferation suggested that the presence of CRNDE would be required for the progression of CRC at physiological levels. Importantly, we also found that overexpression of miR-181a-5p induced the same phenotypes as CRNDE knockdown, which was once again consistent with the inhibitory regulation of miR-181a-5p by CRNDE.

The involvement of miR-181a-5p in cancer development has been studied by many groups, and the results were controversial. MiR-181a-5p was found to be up-regulated in pancreatic cancer and breast cancer [30, 31], and other miR-181 family members were up-regulated in hepatocellular cancer stem cells [32, 33]. However, other studies demonstrated that miR-181a was down-regulated in gliomas [34] and aggressive chronic lymphocytic leukemia [35]. Even in CRC development, miR-181a could function as both oncogene and tumor suppressor. MiR-181a was found to be associated with poor prognosis of colorectal cancer [36, 37]. MiR-181a also promoted tumor growth and liver metastasis in colorectal cancer by targeting the tumor suppressor WIF-1 [38]. In contrast, the expression levels of miR-181a were shown to increase during the progression from non-neoplasia to dysplasia in inflammatory bowel disease-associated CRC, whereas decrease when dysplasia develops into cancer [39]. Our study appears to support a tumor suppressor role for miR-181a-5p, in terms of cell proliferation and chemoresistance. The mechanism of action of miR-181a-5p was initially investigated via target prediction. We identified β-catenin and TCF4 as inhibitory targets of miR-181a-5p. This prediction was confirmed by multiple lines of evidence. First, miR-181a-5p was able to inhibit the wild type 3′-UTR activities of β-catenin and TCF4 in a luciferase assay, but had no effect in the same assay on the activities of the mutant 3′-UTR devoid of miR-181a-5p binding ability. Second, miR-181a-5p overexpression reduced the expression of endogenous β-catenin and TCF4, and thereby inihibited the activity of Wnt/β-catenin signaling.

Although our study did not provide direct evidence whether the changes in Wnt/β-catenin signaling activity were required for the regulation of CRC cell proliferation and chemoresistance by CRNDE and miR-181a-5p, numerous previous studies have implicated Wnt/β-catenin signaling in the regulation of cell proliferation and chemoresistance (summarized in [40]). In this context, it is reasonable for us to conclude that CRNDE promotes CRC cell proliferation and chemoresistance via miR-181a-5p mediated regulation of Wnt/β-catenin signaling. From clinical characteristics analysis, high CRNDE leads to more Lymph node metastasis. Further investigation for CRNDE relative to CRC metastasis is needed.

## Conclusions

In our investigation, we have established a signaling cascade involving CRNDE and miR-181a-5p in the regulation of Wnt/β-catenin signaling, cell proliferation and the chemoresistance of CRC cells. We postulated that if restoration of increased miR-181a-5p expression levels by a miRNA inhibitor could abolish the phenotypes induced by CRNDE knockdown, it would indicate that miR-181a-5p was required for CRNDE to regulate Wnt/β-catenin signaling, CRC cell proliferation and chemoresistance. Our study maybe provide an efficient therapeutic approach for CRC treatments.

## Additional files

Additional file 1: Nuclear location of CRNDE in CRC cancer cells. RNA FISH assays were performed in HCT116 and SW480 cells for CRNDE expression. (TIF 2436 kb)
Additional file 2: Effects of miR-181a-5p on CRNDE expression. **a** Expression levels of miR-181a-5p as determined by qRT-PCR in HCT116 and SW480 cells transfected with miR-181a-5p or miR-control. **b** Expression levels of miR-181a-5p as determined by qRT-PCR in HCT116 and SW480 cells transfected with a microRNA inhibitor targeting miR-181a-5p (miR-181a-5p inhibitor) or a control microRNA inhibitor (miR-control inhibitor). **c** Expression levels of CRNDE as determined by qRT-PCR in HCT116 and SW480 cells transfected with miR-181a-5p or miR-control. **d** Expression levels of CRNDE as determined by qRT-PCR in HCT116 and SW480 cells transfected with a microRNA inhibitor targeting miR-181a-5p or miR-control inhibitor. ***P* < 0.01, ****P* < 0.001. (TIF 2575 kb)
Additional file 3: CRNDE overexpression and miR-181a-5p knockdown promote CRC cell proliferation. **a** MTT cell proliferation assay performed in HCT116 and SW480 cells transfected with plasmids overexpressing CRNDE or a control vector. **b** MTT cell proliferation assay performed in HCT116 and SW480 cells transfected with miR-181a-5p inhibitor or miR-control inhibitor. **P* < 0.05. (TIF 1506 kb)
Additional file 4: CRNDE overexpression and miR-181a-5p knockdown promote CRC cell chemoresistance. **a** MTT cell proliferation assay performed in HCT116 and SW480 cells transfected with plasmids overexpressing CRNDE or a control vector and treated with the indicated concentrations of 5-Fu. **b** MTT cell proliferation assay performed in HCT116 and SW480 cells transfected with miR-181a-5p inhibitor or miR-control inhibitor and treated with the indicated concentrations of 5-Fu. **c** MTT cell proliferation assay performed in HCT116 and SW480 cells transfected with plasmids overexpressing CRNDE or a control vector and treated with the indicated concentrations of Oxa. **d** MTT cell proliferation assay performed in HCT116 and SW480 cells transfected with miR-181a-5p inhibitor or miR-control inhibitor and treated with the indicated concentrations of Oxa. **P* < 0.05. (TIF 2723 kb)
Additional file 5: Inhibition of miR-181a-5p promotes Wnt/β-catenin signaling. **a** Luciferase activity assay performed in HEK293 cells co-transfected with a microRNA inhibitor targeting miR-181a-5p and luciferase reporter plasmids driven by either WT or MUT 3′-UTR of β-catenin and TCF4 that was devoid of miR-181a-5p binding activity. **b** The protein levels of β-catenin and TCF4 as determined by Western blot analysis in HCT116 and SW480 cells transfected with miR-181a-5p inhibitor or miR-control inhibitor. **c** TOP/FOP luciferase activity in HCT116 and SW480 cells transfected with a microRNA inhibitor targeting miR-181a-5p or a control microRNA inhibitor. **P* < 0.05. (TIF 2515 kb)
Additional file 6: Wnt/β-catenin signaling is required for CRC cell chemoresistance. **a** β-catenin was silenced in HCT116 and SW480 cells. The protein levels of β-catenin as determined by Western blot analysis. **b** TCF4 was deleted in HCT116 and SW480 cells. The protein levels of TCF4 as determined by Western blot analysis. **c** MTT cell proliferation assay performed in β-catenin silencing HCT116 and SW480 cells treated with the indicated concentrations of 5-Fu. **d** MTT cell proliferation assay performed in TCF4 deletion HCT116 and SW480 cells treated with the indicated concentrations of 5-Fu. **e** MTT cell proliferation assay performed in β-catenin silencing HCT116 and SW480 cells treated with the indicated concentrations of Oxa. **f** MTT cell proliferation assay performed in TCF4 deletion HCT116 and SW480 cells treated with the indicated concentrations of Oxa. **P* < 0.05. (TIF 3961 kb)
Additional file 7: Regulation of CRC cell proliferation, chemoresistance and Wnt/β-catenin signaling by CRNDE requires miR-181a-5p. **a** MTT cell proliferation assay, **b** colony formation assay, **c** MTT cell proliferation assay under the treatment of the indicated concentrations of 5-Fu or **d** Oxa, **e** The protein levels of β-catenin and TCF4 as determined by Western blot analysis, and **f** TOP/FOP luciferase activity assay performed in SW480 cells transfected with siRNA targetting CRNDE or siR-control simultaneously with miR-181a-5p or miR-control. **P* < 0.05. (TIF 4563 kb)

## Abbreviations

5-Fu: 5-fluorouracil
CRC: Colorectal cancer
CRNDE: Colorectal neoplasia differentially expressed
HUVEC: Human umbilical vein endothelial cells
lncRNAs: Long noncoding RNAs
ncRNAs: Noncoding RNAs
Oxa: Oxaliplatin
TCGA: The cancer genome atlas
UTR: Untranslated region
