# Supplementary figures and images for: The lncRNA CRNDE promotes colorectal cancer cell proliferation and chemoresistance via miR-181a-5p-mediated regulation of Wnt/β-catenin signaling
Source: Mol Cancer. 2017 Jan 13;16:9. doi: 10.1186/s12943-017-0583-1 (PMC5237133; doi:10.1186/s12943-017-0583-1)

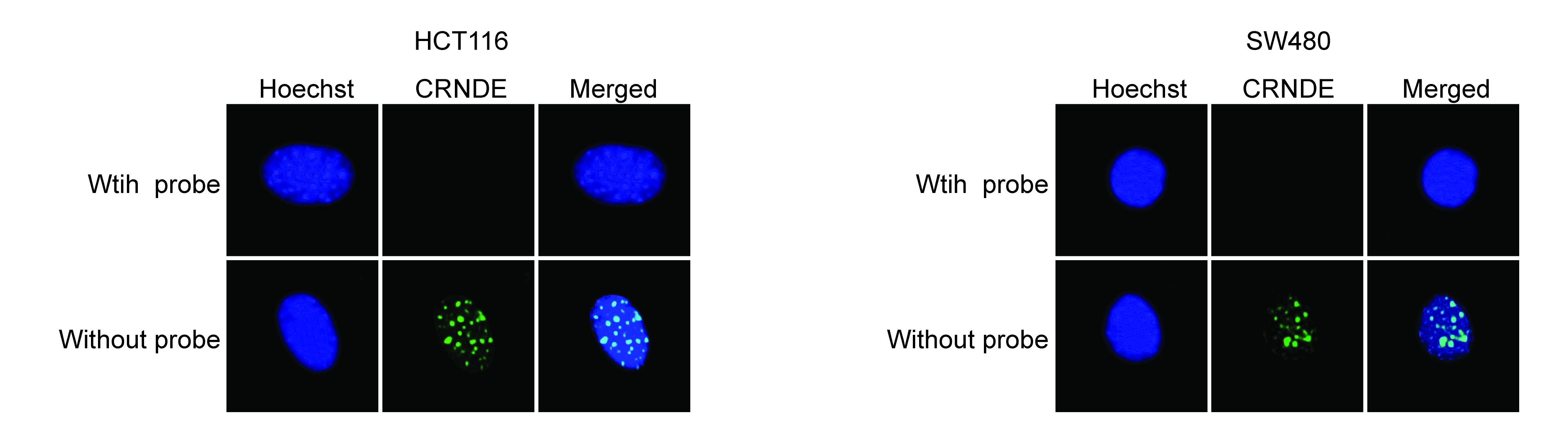

Supplement: Additional file 1: — Nuclear location of CRNDE in CRC cancer cells. RNA FISH assays were performed in HCT116 and SW480 cells for CRNDE expression. (TIF 2436 kb) [file 12943_2017_583_MOESM1_ESM.tif]

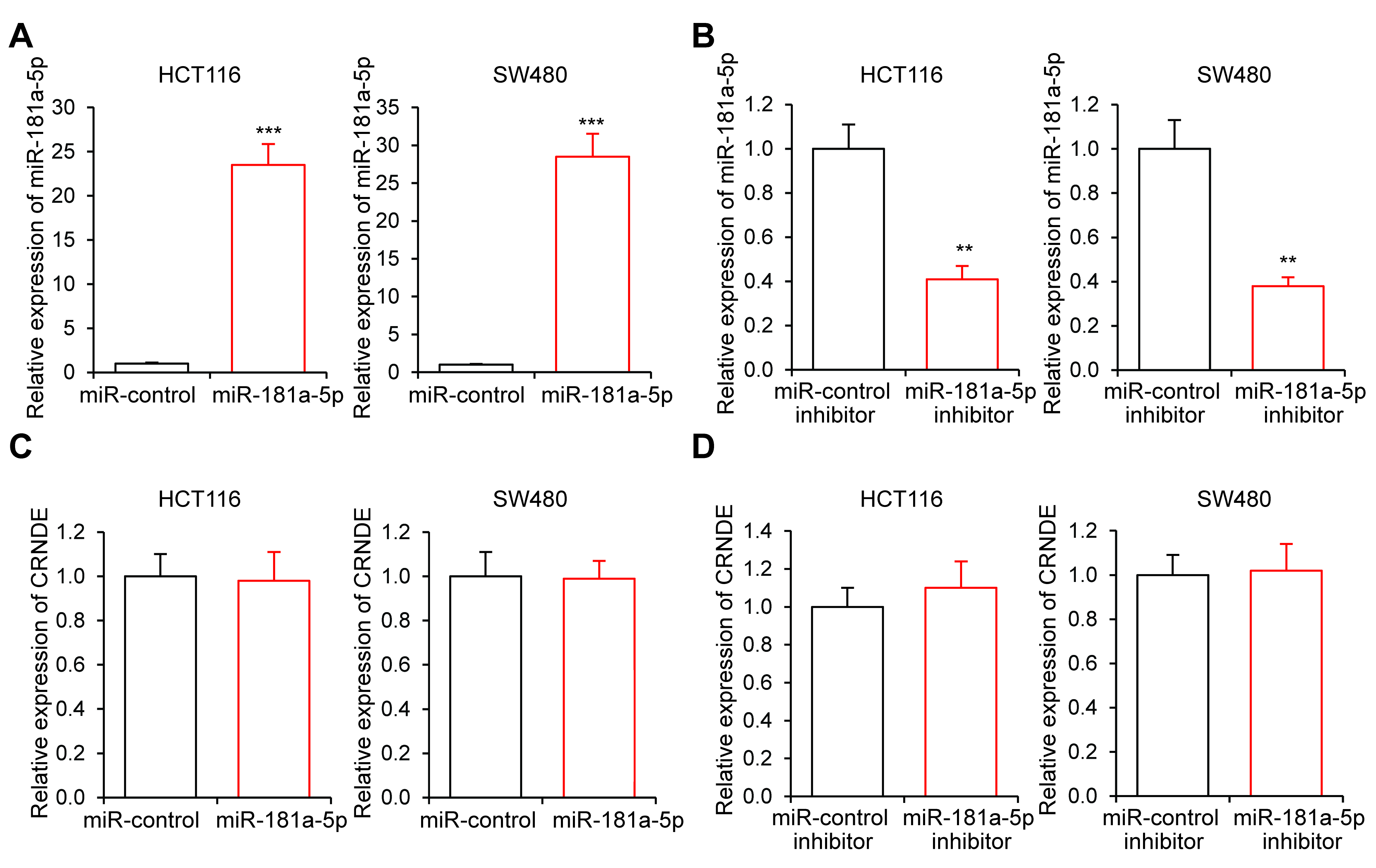

Supplement: Additional file 2: — Effects of miR-181a-5p on CRNDE expression. a Expression levels of miR-181a-5p as determined by qRT-PCR in HCT116 and SW480 cells transfected with miR-181a-5p or miR-control. b Expression levels of miR-181a-5p as determined by qRT-PCR in HCT116 and SW480 cells transfected with a microRNA inhibitor targeting miR-181a-5p (miR-181a-5p inhibitor) or a control microRNA inhibitor (miR-control inhibitor). c Expression levels of CRNDE as determined by qRT-PCR in HCT116 and SW480 cells transfected with miR-181a-5p or miR-control. d Expression levels of CRNDE as determined by qRT-PCR in HCT116 and SW480 cells transfected with a microRNA inhibitor targeting miR-181a-5p or miR-control inhibitor. **P < 0.01, ***P < 0.001. (TIF 2575 kb) [file 12943_2017_583_MOESM2_ESM.tif]

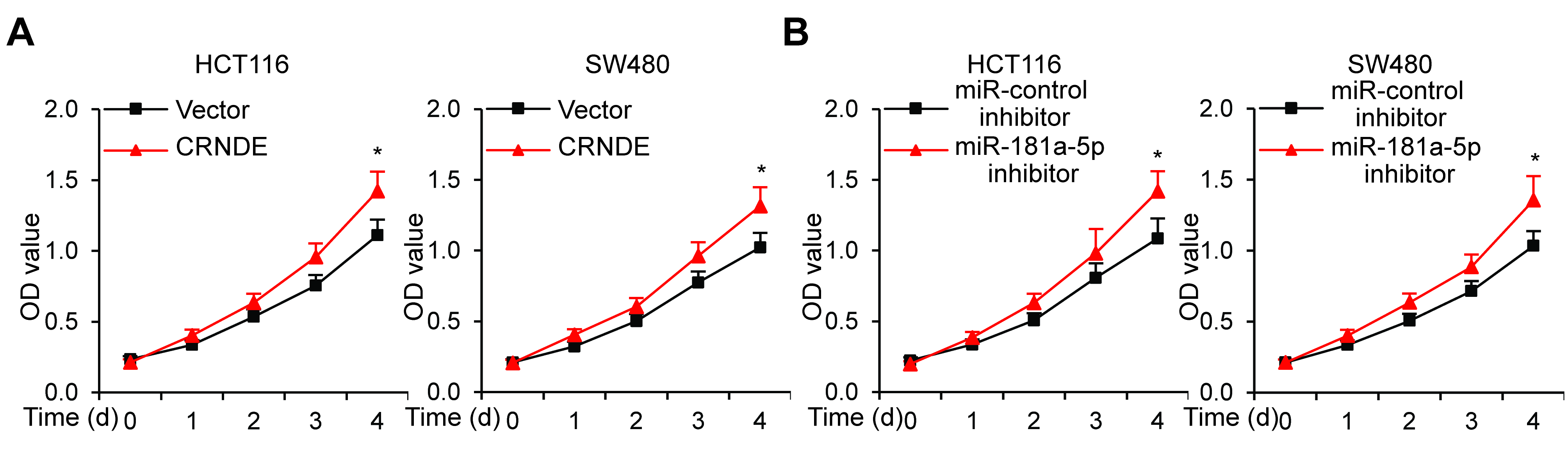

Supplement: Additional file 3: — CRNDE overexpression and miR-181a-5p knockdown promote CRC cell proliferation. a MTT cell proliferation assay performed in HCT116 and SW480 cells transfected with plasmids overexpressing CRNDE or a control vector. b MTT cell proliferation assay performed in HCT116 and SW480 cells transfected with miR-181a-5p inhibitor or miR-control inhibitor. *P < 0.05. (TIF 1506 kb) [file 12943_2017_583_MOESM3_ESM.tif]

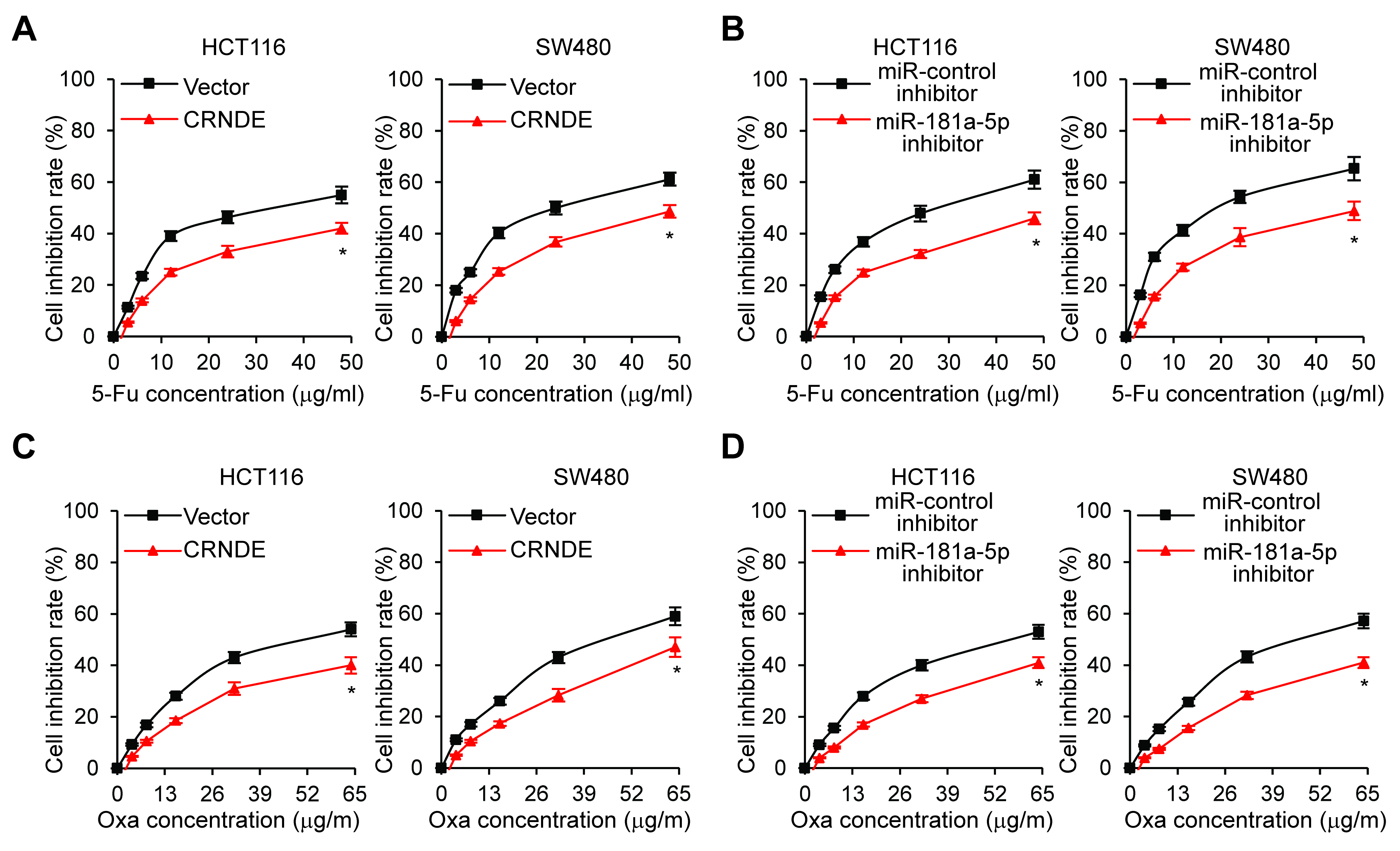

Supplement: Additional file 4: — CRNDE overexpression and miR-181a-5p knockdown promote CRC cell chemoresistance. a MTT cell proliferation assay performed in HCT116 and SW480 cells transfected with plasmids overexpressing CRNDE or a control vector and treated with the indicated concentrations of 5-Fu. b MTT cell proliferation assay performed in HCT116 and SW480 cells transfected with miR-181a-5p inhibitor or miR-control inhibitor and treated with the indicated concentrations of 5-Fu. c MTT cell proliferation assay performed in HCT116 and SW480 cells transfected with plasmids overexpressing CRNDE or a control vector and treated with the indicated concentrations of Oxa. d MTT cell proliferation assay performed in HCT116 and SW480 cells transfected with miR-181a-5p inhibitor or miR-control inhibitor and treated with the indicated concentrations of Oxa. *P < 0.05. (TIF 2723 kb) [file 12943_2017_583_MOESM4_ESM.tif]

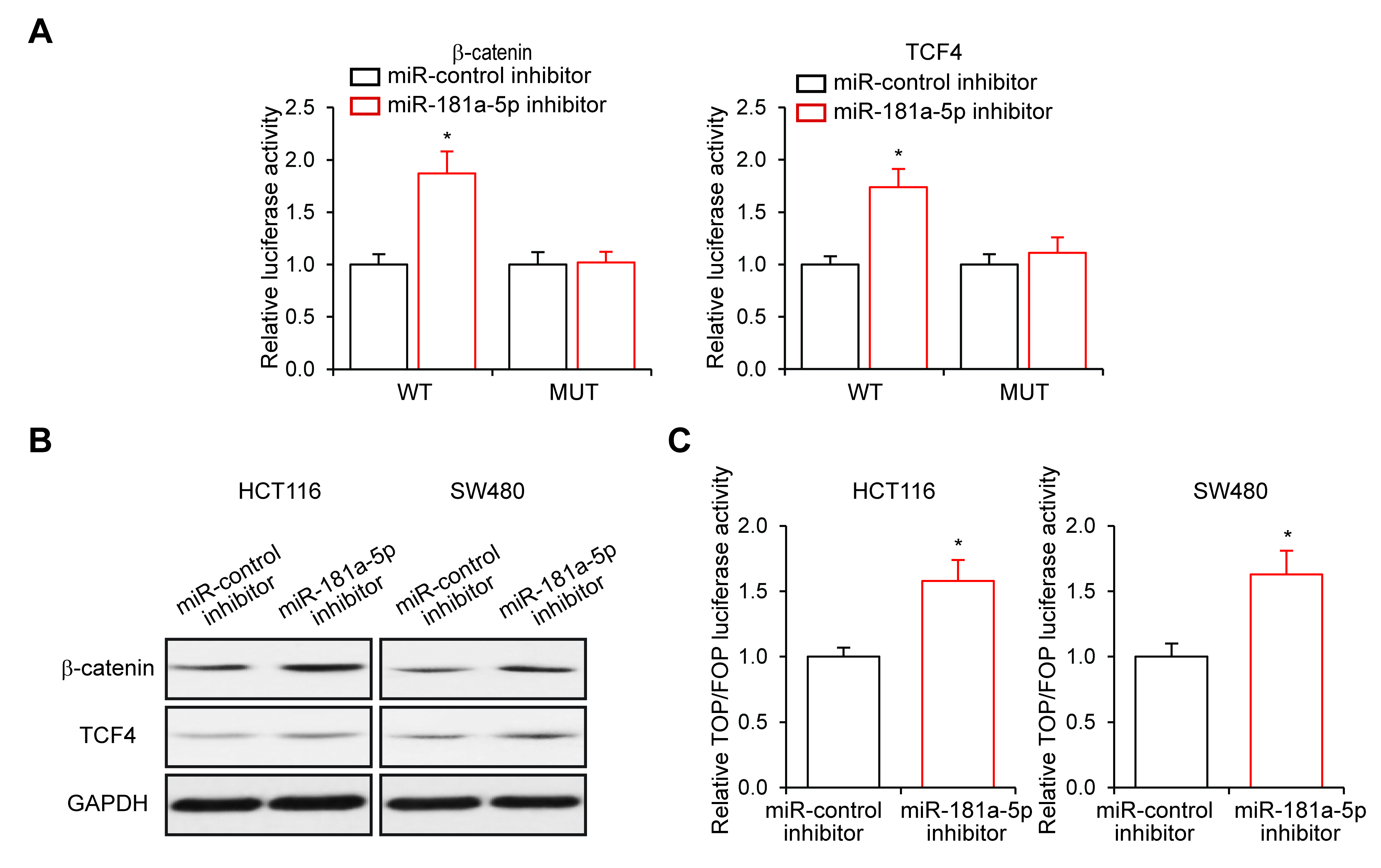

Supplement: Additional file 5: — Inhibition of miR-181a-5p promotes Wnt/β-catenin signaling. a Luciferase activity assay performed in HEK293 cells co-transfected with a microRNA inhibitor targeting miR-181a-5p and luciferase reporter plasmids driven by either WT or MUT 3′-UTR of β-catenin and TCF4 that was devoid of miR-181a-5p binding activity. b The protein levels of β-catenin and TCF4 as determined by Western blot analysis in HCT116 and SW480 cells transfected with miR-181a-5p inhibitor or miR-control inhibitor. c TOP/FOP luciferase activity in HCT116 and SW480 cells transfected with a microRNA inhibitor targeting miR-181a-5p or a control microRNA inhibitor. *P < 0.05. (TIF 2515 kb) [file 12943_2017_583_MOESM5_ESM.tif]

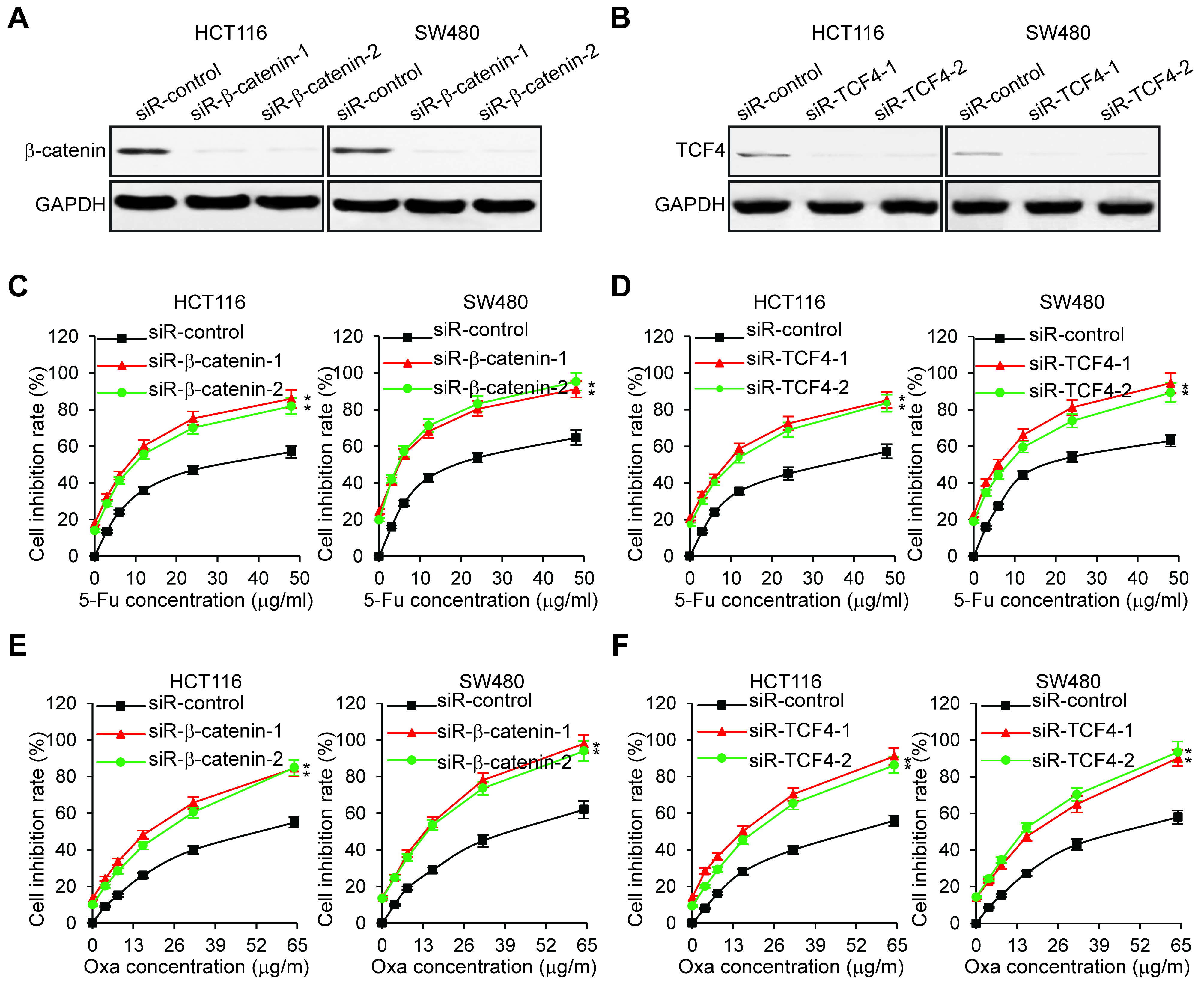

Supplement: Additional file 6: — Wnt/β-catenin signaling is required for CRC cell chemoresistance. a β-catenin was silenced in HCT116 and SW480 cells. The protein levels of β-catenin as determined by Western blot analysis. b TCF4 was deleted in HCT116 and SW480 cells. The protein levels of TCF4 as determined by Western blot analysis. c MTT cell proliferation assay performed in β-catenin silencing HCT116 and SW480 cells treated with the indicated concentrations of 5-Fu. d MTT cell proliferation assay performed in TCF4 deletion HCT116 and SW480 cells treated with the indicated concentrations of 5-Fu. e MTT cell proliferation assay performed in β-catenin silencing HCT116 and SW480 cells treated with the indicated concentrations of Oxa. f MTT cell proliferation assay performed in TCF4 deletion HCT116 and SW480 cells treated with the indicated concentrations of Oxa. *P < 0.05. (TIF 3961 kb) [file 12943_2017_583_MOESM6_ESM.tif]

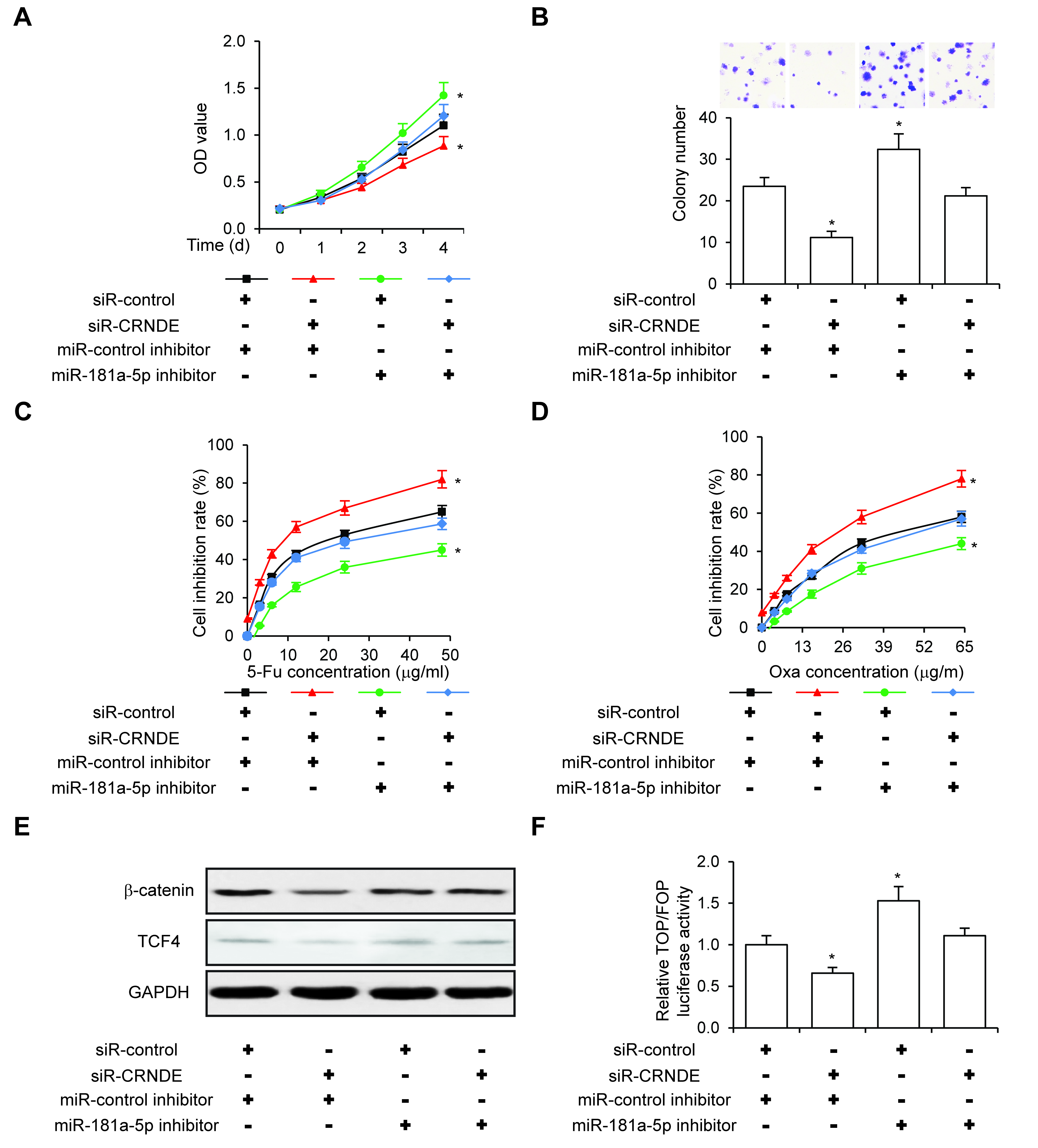

Supplement: Additional file 7: — Regulation of CRC cell proliferation, chemoresistance and Wnt/β-catenin signaling by CRNDE requires miR-181a-5p. a MTT cell proliferation assay, b colony formation assay, c MTT cell proliferation assay under the treatment of the indicated concentrations of 5-Fu or d Oxa, e The protein levels of β-catenin and TCF4 as determined by Western blot analysis, and f TOP/FOP luciferase activity assay performed in SW480 cells transfected with siRNA targetting CRNDE or siR-control simultaneously with miR-181a-5p or miR-control. *P < 0.05. (TIF 4563 kb) [file 12943_2017_583_MOESM7_ESM.tif]
